# Determinants of HIV outpatient service utilization according to HIV parameters

**DOI:** 10.7448/IAS.17.4.19611

**Published:** 2014-11-02

**Authors:** Paola Di Carlo, Palmira Immordino, Giovanni Mazzola, Pietro Colletti, Ilenia Alongi, Maurizio Mineo, Marco Scognamillo, Francesco Vitale, Alessandra Casuccio

**Affiliations:** 1 Department of Sciences for Health Promotion and Mother-Child Care, University of Palermo, Palermo, Italy; 2 Infectious Disease Unit, University Hospital “Paolo Giaccone”, Palermo, Italy

## Abstract

**Introduction:**

The increased life expectancy of HIV patients in the era of highly active antiretroviral therapy has had profound consequences for the healthcare systems that provide their care. It is useful to assess whether healthcare resources need to be adapted to the different stages of HIV infection or to patient characteristics [1]. To study how patient features influence utilization of out patient services, we retrospectively analyzed the electronic health record of HIV-positive patients who had followed day-care programs at the AIDS Center of the University of Palermo, Italy.

**Materials and Methods:**

223 HIV-infected subjects were recruited and divided into two groups according to CD4 cell counts (117 with a CD4 count ≤500/mm^3^ and 106 with CD4 count ≥500/mm^3^). Data on age, gender, race, lifestyle habits (including educational level, drug abuse history, smoking status, alcohol consumption, sexual behaviour) BMI, HIV-RNA, CD4+ T-cell count, antiretroviral therapy (ART), comorbidities such as HCV co-infection, osteoporosis biomarker, dyslipidemia, diabetes, renal function and systolic and diastolic blood pressure were recorded in a purposely designed database and were analyzed in relation to AIN by uni- and multivariable logistic regression.

**Results:**

Table 1 shows the characteristics of enrolled patients; the average age of the recruited patients was 45.4±9.5 years. 163 individuals were male (73%), 26 were immigrants (12%) and 91 (40%) were treatment-naïve. Mean day care access for laboratory tests to evaluate stage of HIV and for treatment monitoring was 6.5 days for CD4 cell count measurements and 9.6 for HIV RNA/drug-resistance testing. When patients were stratified according to CD4 count, mean day care access for laboratory tests to evaluate HIV stage and to monitor treatment was negatively correlated with CD4 cell counts.

**Conclusions:**

Only patients with CD4 counts ≤500/mm^3^ showed higher rates of healthcare utilization; these data may be useful for monitoring and revising implementation plans for the different phases of HIV disease.

**Table 1:** Selected characteristics of 223 HIV-infected patients

Variable	HIV with a CD4 count ≤500/mm^3^ n = 117	HIV with a CD4 count ≥500/mm^3^ n = 106	p
Age years (mean and SD)	45.4	45.8	ns
Male/Female	84/33	79/27	ns
BMI (kg/m^2^) (mean and SD)	23.4	24.6	ns
Ethnicity (n) Caucasian /African	99/18	98/8	ns
High-risk sexual behaviors Heterosex/homosex/bisexual men	20/29/10	35/22/20	ns
CD4+ T-cell count (cells/∣ÌL) (mean)	310	1044	///
HIV-RNA copies/mL (mean)	25.728	6.669	ns
25-Hydroxyvitamin D (ng/mL)	28.9	25.1	ns
Drug addiction (n)	33	22	ns
ARV therapy (<5 years)	52	30	0.014
ARV therapy (five-ten years)	17	20	ns
ARV therapy (>10 years)	45	52	ns
Mean day care access for laboratory tests CD4/Viral load	7.0/10.1	6.2/9.4	0.045/ns
